# Seeing beyond a Dilated Proventriculus: Diagnostic Tools for Proventricular Dilatation Disease in Psittacine Birds

**DOI:** 10.3390/ani11123558

**Published:** 2021-12-14

**Authors:** Jeann Leal de Araújo, Raquel Rubia Rech

**Affiliations:** 1 Department of Veterinary Sciences, Center for Agricultural Sciences, Federal University of Paraíba, Areia 58397000, Paraiba, Brazil; 2 Department of Veterinary Pathobiology, Texas A&M University, College Station, TX 77843-4467, USA; RRech@cvm.tamu.edu

**Keywords:** PDD, encephalitis, avian diseases, wildlife, parrots, ex situ conservation

## Abstract

**Simple Summary:**

Proventricular dilatation disease (PDD) is a life-threatening neurological disease that affects several psittacine species, including endangered ones. Recognizing which tools are available and which ones are more appropriate for different antemortem and postmortem situations is crucial for an effective and quick diagnosis. Here, we review the main diagnostic strategies for PDD in psittacine birds, which can help veterinary clinicians and diagnosticians to offer the best approaches regarding diagnostic-oriented biotechnologies for pet owners, zoos, rehabilitation centers, breeding facilities and other institutions working with ex situ avian conservation. A combination of different strategies can increase the success of diagnostic outcomes.

**Abstract:**

Proventricular dilatation disease (PDD) is a life-threatening neurological disease caused by parrot bornaviruses (PaBVs) that affects several species worldwide. PDD can be clinically manifested as either a central nervous system condition or a gastrointestinal condition if the nerves and ganglia of the gastrointestinal tract are compromised. We intend to provide a concise review for veterinary clinicians and diagnosticians with focus on the main tools available for PDD diagnosis, including gross and histopathology, immunohistochemistry, molecular techniques and serology. We suggest that a combination of different strategies can increase the success of diagnostic outcomes, as tools such as reverse transcription polymerase chain reaction (RT-PCR) and enzyme-linked immunosorbent assay (ELISA) can be implemented for identification of bornaviral infections in live patients, and gross pathology, histopathology, immunohistochemistry and RT-PCR can provide reliable results for postmortem diagnosis of PDD.

## 1. Introduction

**Figure 1:**
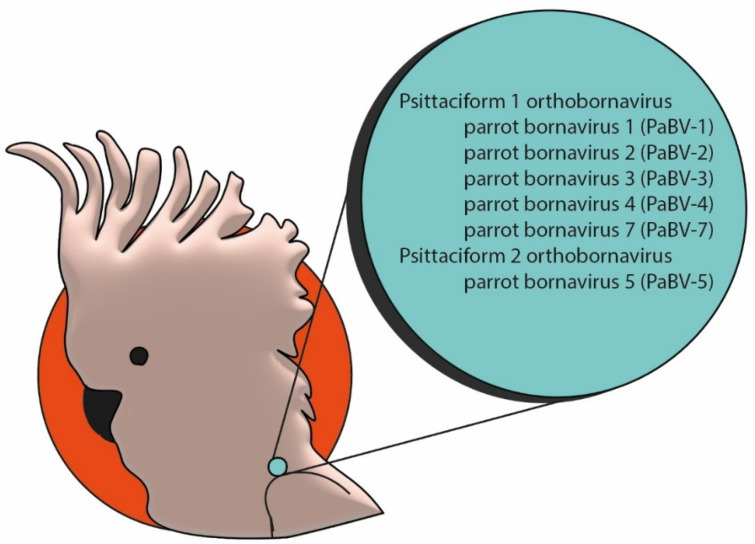
Bornaviruses specific to psittacine birds described to date.

Proventricular dilatation disease (PDD) is a life-threatening neurological condition that affects captive psittacine birds worldwide. Although initially described in macaws in the 1970s, its causative agent remained unknown for decades [1,2]. In 2008, two independent research groups identified that the etiologic agent of PDD belonged to the family Bornaviridae [3,4]. After this discovery, several studies experimentally induced classic clinical signs and lesions of PDD in psittacine birds after inoculation of bornaviruses recovered from naturally affected birds, supporting the causal relationship of bornaviruses and PDD [5,6,7,8,9,10,11]. Bornaviruses are negative-sense simple-stranded RNA viruses that belong to the order Mononegavirales. This order underwent several taxonomic modifications in the last few years due to the discovery of new viruses of birds, reptiles, mammals and fish [12,13]. The family Bornaviridae now encompasses three genera: Carbovirus, Cultervirus and Orthobornavirus. The latter comprises viral species that affect mammals, reptiles and birds. Five of these virus species affect only avian species: Passeriform 1 bornavirus, Passeriform 2 orthobornavirus, Waterbird 1 orthobornavirus, Psittaciform 1 orthobornavirus and Psittaciform 2 orthobornavirus. Several genotypes including parrot bornaviruses (PaBV)-1, 2, 3, 4, 5, 6 and 7, which belong to the species Psittaciform 1 and 2 orthobornavirus, have been identified in psittacine birds [12], and are summarized in Figure 1.

Some studies have proposed the possibility of an autoimmune component to be involved in the development of PDD lesions and question if other causative agents can trigger these lesions [14,15]. To date, the only pathogens that have been extensively and consistently identified, through immunohistochemical and molecular methods in natural and experimental cases of PDD, are PaBVs. Additional studies are needed to understand molecular aspects of tissue injury and lesion development. It has also been suggested that the term “avian ganglioneuritis (AG)” rather than PDD, should be used [15]. However, since the central nervous system can also be affected by lymphoplasmacytic infiltrates, this nomenclature does not reflect the complete spectrum of lesions that can be observed in PDD cases. PDD has been reported in several psittacine species kept in captivity in South and North America, Africa, Oceania and Asia, including Amazon parrots (*Amazona* spp.), African grey parrots (*Psittacus* spp.), macaws (e.g., *Ara* spp., *Cyanopsitta spixii*, etc.), cockatoos (*Cacatuidae*), cockatiels (*Nymphicus hollandicus*), conures (e.g., *Aratinga* spp., *Cyanoliseus* spp., etc.), and budgerigars (*Melopsittacus undulatus).* From the point of view of conservation, this disease has raised concern especially for critically endangered species participating in ex situ conservation programs, such as in the case of the Spix’s macaw (*Cyanopsitta spixii*), which is currently extinct in the wild [16].

Several aspects of PDD remain to be elucidated, including routes of transmission, molecular mechanisms of injury and immunopathology. However, the causal relationship of PaBVs and PDD has been extensively documented and several techniques can be implemented in the correct diagnosis of this important disease. In this review, we explore the main diagnostic tools that can be used for the diagnosis of PDD, with focus on ex situ conservation.

## 2. Diagnostic Techniques

### 2.1. Clinical Evaluation

Recognizing clinical signs associated with PDD is crucial for the clinical outcome of potentially PaBV-infected birds. Although the name of the disease implies a gastrointestinal presentation, PDD is, in fact, a misnomer, and it is primarily a neurological disease. PaBVs can affect either the central nervous system (CNS) or the peripheral nervous system (PNS). When the CNS is affected by PaBVs, PDD manifests as an encephalomyelitis with clinical signs that might include ataxia, seizures, paresis, paralysis, lethargy, obtundation, somnolence and coma. When the PNS is affected, the lesion is a ganglioneuritis, and clinical signs will vary according to the location of the compromised innervation. Most often, the enteric nervous system (ENS) is affected, which leads to the onset of gastrointestinal signs, such as undigested seeds in the feces, regurgitation, emaciation, cachexia and the classic dilated proventriclus [17]. Thus, PDD clinical signs are highly variable, and studies have indicated that different PaBVs can induce different patterns of lesions and consequently clinical disease, with predominantly neurological or gastrointestinal signs [5]. A study conducted in Brazil revealed that out of 32 PaBV-positive psittacine birds, 66% presented with neurological signs, 22% presented with gastrointestinal signs and approximately 9% had a history of sudden death [18].

Furthermore, it has been recently documented that sudden death due to cardiac ganglioneuritis and myocarditis might be an important clinical manifestation of PDD [19].

### 2.2. Imaging Techniques

**Figure 2:**
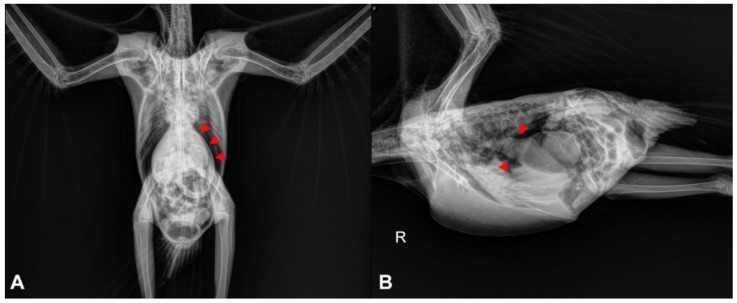
Radiographic projections of a young macaw with PDD. (**A**) Ventrodorsal projection. Moderate dilatation of the proventriculus (arrowheads). The proventriculus extends beyond the liver edge. (**B**) Lateral projection. The proventriculus (arrowheads) is moderately distended and contains ingesta and gas. R = Right side.

In cases of suspected gastrointestinal disorders related to PDD, diagnostic imaging may be a useful resource for the clinician. The most cost-effective imaging tools are usually survey and contrast radiographs, which are often readily available for clinical veterinarians (Figure 2). These techniques can provide relevant information about the size and motility of the gastrointestinal compartments, especially the proventriculus [20].

It has been suggested that proventricular diameter-to-keel height ratio obtained from lateral radiographic projections is a valuable method for identification of an enlarged proventriculus, and ratio values above 0.52 indicate proventricular dilatation [21]. It is important to note that PDD is not the only disease that can cause proventricular dilatation in psittacine birds, and other causes such as foreign bodies, neoplasia, lead toxicity and *Macrorhabdus ornithogaster* infection should be ruled out. 

In healthy psittacine birds, contrast media such as barium sulfate should be visualized by radiographs reaching the cloaca between 90 min to 3 h after its administration by gavage [22]. Therefore, contrast radiographs may be useful to identify alterations in the gastrointestinal transit time, and it has been observed that PDD-affected birds may have an increased transit time [20].

Other imaging techniques such as fluoroscopy can also be implemented for evaluation of the gastrointestinal tract of PDD-suspected birds; however, this a more expensive diagnostic resource and requires costly equipment [20]. Due to the possibility of cardiac disease induced by PDD, cardiac imaging techniques such as an echocardiogram might be a useful technique. It has been documented that an echocardiogram of a case of naturally occurring PDD revealed tachycardia, arrythmia, pericardial effusion, ventricular dilatation, decreased ventricular fractional shortening and mitral regurgitation [19].

### 2.3. Gross Pathology

**Figure 3:**
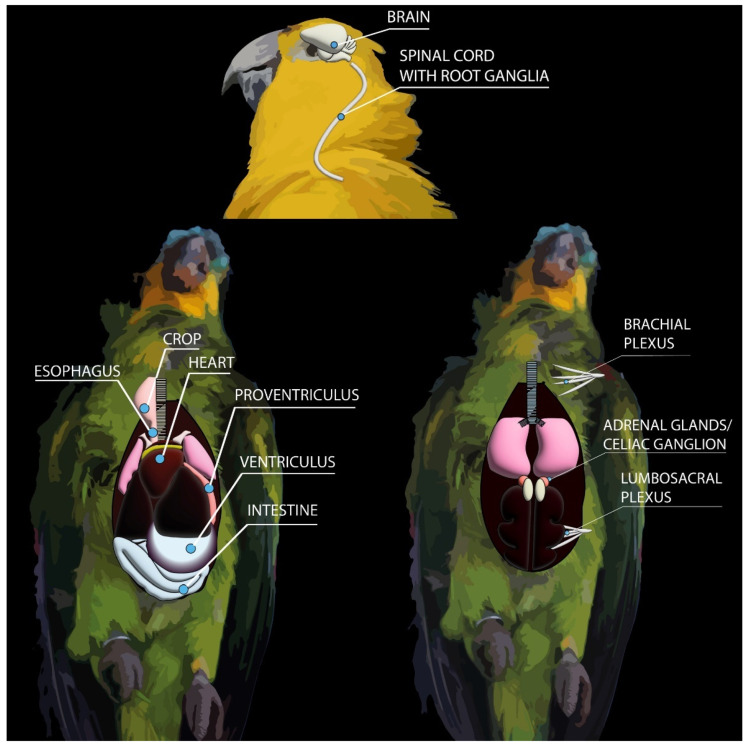
Key tissues for assessment and sampling during necropsies of PDD-suspected psittacine birds. These are the main organs that should be investigated using histopathology, immunohistochemistry and RT-PCR for confirmation of PDD.

The first studies that contributed to the diagnosis of PDD in psittacine birds were published in the 1980s [2] and relied mostly on gross and histopathological changes observed on necropsied birds. These diagnostic techniques can consistently identify key morphologic changes that are characteristic of PDD, and still remain the fundamental tools for a highly suggestive diagnosis and can guide the diagnostician when ancillary testing is needed. Figure 3 illustrates the main organs that should be assessed during necropsy and collected for further histopathological, immunohistochemical and molecular analysis in suspected cases of PDD.

**Figure 4:**
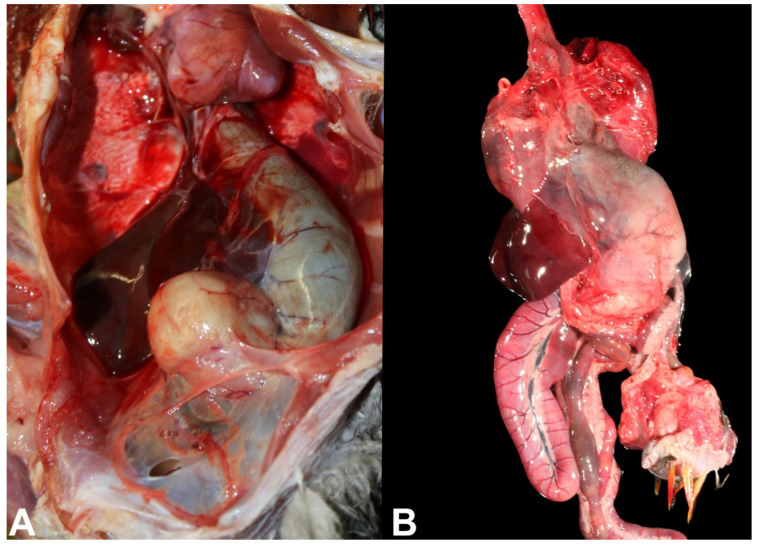
Macroscopic lesions of PDD in psittacine birds. (**A**) Thin-walled distended proventriculus in the coelomic cavity of a red-and-green macaw hybrid (*Ara chloropterus*). (**B**) Ex situ thin-walled distended proventriculus of a Catalina macaw.

The most striking macroscopic finding in birds affected by PDD is a distended proventriculus (Figure 4A,B) [2,23] that is usually accompanied by marked thinning of the proventricular wall, which in severe cases, can rupture [24]. Similarly, if crop innervation is compromised, a dilated crop can also be identified in PDD birds, and it is usually associated with regurgitation [10]. However, not all birds will develop proventricular dilatation as they may have only the CNS affected, and, therefore, will only manifest central neurological signs. In these cases, no gross lesions are observed in the brain or spinal cord.

Due to the progressive and chronic nature that usually characterizes PDD, cachexia is frequently observed due to impairment gastrointestinal motility. Consequently, carcasses of birds affected by PDD typically have poor body condition scores. Cardiomegaly and/or hydropericardium can be observed in birds with sudden death [19].

During necropsy of a PDD-suspected bird, sections of all tissues must be collected in 10% neutral-buffered formalin for further histopathological processing. Additionally, it is recommended for spinal cord sampling that the vertebral bodies should not be separated from the spinal cord; rather, transverse sections of the vertebral bodies and spinal cord should be obtained together and fixed in formalin, as this process preserves the dorsal and ventral root ganglia in the sample [25]. 

### 2.4. Histopathology

**Figure 5:**
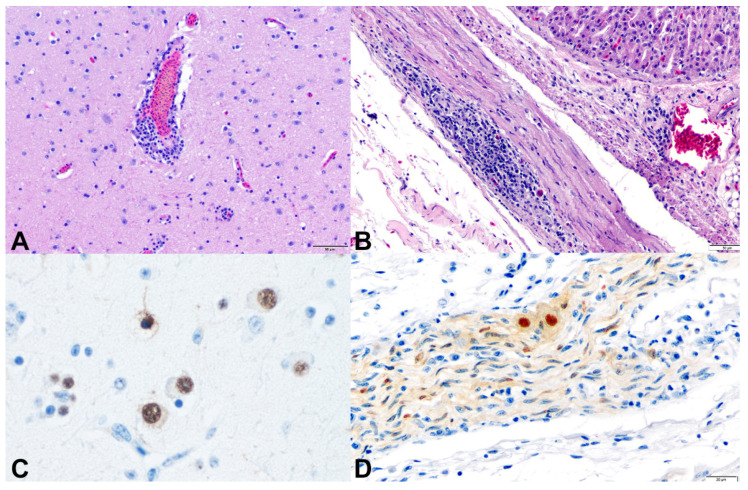
Histopathological and immunohistochemical features of PDD in psittacine birds. (**A**) Lymphoplasmacytic encephalitis. Brain. *Ara chloropterus*. (**B**) Lymphoplasmacytic ganglioneuritis. Proventriculus. *Cacatua alba.* (**C**) Intranuclear immunolabeling for PaBV-2 in neurons. Brain. *Cacatua alba*. (**D**) Intranuclear and intracytoplasmic immunolabeling for PaBV-2 in neurons of an epicardial ganglion. *Psittacus erithacus*.

Microscopically, lesions of PDD are predominantly characterized by inflammatory infiltrates composed of lymphocytes, plasma cells and fewer macrophages in the CNS and PNS. Rarely Mott cells can be observed [26]. Eosinophilic intranuclear inclusions in neurons, a typical feature observed in mammals with Borna disease, is not observed in birds [25]. In the CNS, the inflammatory infiltrates are usually represented by perivascular cuffs (Figure 5A) that can be observed in the brain or in the spinal cord, affecting primarily the grey matter. These infiltrates can also affect the meninges. In a timeline experimental study, perivascular cuffs were more frequently observed in the most caudal–ventral areas of the brain, with higher occurrence in the thalamus and hindbrain [10].

PDD lesions in the PNS are represented by lymphoplasmacytic infiltrates (Figure 5B) that affect nerves and ganglia of a wide range of organs, most commonly the crop, proventriculus, ventriculus, small intestine, heart, celiac ganglion and adrenal glands. Based on the identification of inflammatory infiltrates, crop biopsy has been indicated as a potential minimally invasive ante-mortem diagnostic method for PDD. However, the success of this technique relies largely on the presence of ganglia and/or nerves in the collected sample and therefore, false-negative results are common and can reach up to 30% [27]. 

Histopathology is an essential tool for the diagnosis of PDD, since the identification of the lymphoplasmacytic meningoencephalomyelitis with systemic ganglioneuritis are decisive key lesions that can indicate the best ancillary tests that the pathologist can use in order to confirm or rule out PDD.

### 2.5. Immunohistochemistry

Following the first confirmation studies to link PDD and bornaviruses [3,4], immunohistochemical analyses to identify bornaviral antigens in avian tissues were first published in 2009 [28,29,30,31]. Since then, several studies have been able to consistently immunolabel PaBVs in formalin-fixed paraffin-embedded (FFPE) tissues [32,33,34,35], allowing immunohistochemistry (IHC) to become an effective strategy for post-mortem diagnosis of PDD. 

Through IHC, strong intranuclear and mild to moderate intracytoplasmic immunolabeling can be observed in several infected cells, such as neurons in the CNS (Figure 5C) or in the ganglia (Figure 5D). Due to the nuclear replication of PaBVs, cells with only intracytoplasmic immunolabeling should not be considered as a positive result [10]. 

Initially, when specific avian antibodies for PaBVs were not available, antibodies against the nucleoprotein (N) [30] or phosphoprotein (P) [29] protein of Borna disease virus (BoDV) were used for immunohistochemical analysis. However, specific anti-PaBV antibodies were rapidly developed and integrated into the diagnosis and research of PDD, especially those targeting the PaBV N protein [10,28,31,32,33].

Although molecular techniques had already demonstrated that PaBVs could infect not only the gastrointestinal tract of birds, but also the CNS [3], IHC had a crucial role to support this finding and to determine the antigen anatomical distribution of PaBVs in a wide range of tissues, indicating the presence of viral antigen in areas of lymphoplasmacytic inflammation [33]. Nonetheless, IHC has also been an important tool do determine that PaBVs can infect several cells of the CNS, including neurons, glial cells, and ependymal cells [25].

IHC is an essential diagnostic tool for samples collected during necropsy, especially when frozen or fresh tissues are not collected or properly stored. One important drawback of this technique is that anti-PaBV antibodies are not readily available for commercial use and few laboratories have standardized and optimized this technique. 

Histopathology is a useful tool to determine which tissues should be used for immunohistochemical identification of PaBVs as inflammatory infiltrates are often associated with viral antigen. Target tissues include the proventriculus, ventriculus, small intestine, celiac ganglion, adrenal glands and the brain. Regarding different areas of the CNS, under experimental conditions, the thalamus and hindbrain are considered the most appropriate sections for sampling and determination of PaBV-antigen distribution, whereas little or no difference as been observed between the cervical, thoracic and lumbosacral sections of the spinal cord [25]. 

### 2.6. Molecular Techniques

Until 2008, the diagnosis of PDD was based solely on gross and microscopic lesions, and thus confirmation could only be achieved if the patients died or if a crop biopsy containing the affected ganglia or serves was successfully obtained. After the identification of PaBVs as the causative agents of PDD, new diagnostic tools became available, and an ante-mortem diagnosis of PaBV infection could now be performed by analyzing samples using reverse transcriptase polymerase chain reaction (RT-PCR). Recently, a multiplex RT-PCR assay was successfully developed to simultaneously detect multiple avian bornaviruses [36].

Choanal and cloacal swabs are the most common ante-mortem samples used for molecular investigation of PaBVs, but feces and urine have also yielded positive results in some studies. Feather calami and whole blood have been designated by some researchers as reliable samples for molecular diagnosis of PaBVs, although some studies have indicated little or no usefulness of these samples for routine diagnosis of PDD. 

It is important to note the PaBVs are shed intermittently by infected birds and analyzing a single sample taken from a bird at a single timepoint has limited diagnostic utility, as this can lead to a false negative result. Therefore, a negative RT-PCR result does not exclude the possibility of infection. It has been demonstrated that birds with negative RT-PCR results of cloacal and choanal swabs, especially early in the course of infection, have positive results when the brain and spinal cord are analyzed by IHC and RT-PCR [10]. In suspected cases of PDD, it is recommended to acquire samples from different timepoints to reduce the chances of false negative results. 

For samples collected during necropsy, key tissues for RT-PCR include brain, spinal cord, crop, esophagus, proventriculus, ventriculus, small intestine, adrenal glands, heart and nerves (Figure 3). Consequently, during necropsy of PDD-suspected birds, it is important to collect tissue sections not only in 10% neutral-buffered formalin for histopathology/IHC, but also sections that will be frozen and stored at −80 °C for further molecular analysis. As PaBVs are RNA viruses, samples can be collected and placed in RNA stabilization and protection reagents to prevent RNA degradation and to ensure the quality and quantity of the RNA for further molecular analysis. 

### 2.7. Clinical Pathology and Serology

Birds suffering from PDD show inconsistent changes in hematology and clinical chemistry assessments, and most are usually related to the catabolic state induced by gastrointestinal malabsorption, such as nonregenerative anemia and decreased total protein and albumin levels [20,37]. Additionally, elevated levels of muscle enzymes such as lactate dehydrogenase, creatine kinase and aspartate aminotransferase may be seen in infected birds. Other changes such as leukocytosis and heterophilia have been inconsistently reported in some patients and may be associated with stress and secondary infections [20]. These findings have been supported by experimental studies [37]. Although the bloodwork of patients suffering from PDD might not indicate any definitive indication of the infection, it is advisable to perform hematology and clinical chemistry assessments of suspected patients to rule out differential diagnoses and to assess their general health.

Birds mount a detectable, although variable, humoral response to bornaviral infections. Therefore, serological methods such as Western blotting and enzyme-linked immunosorbent assay (ELISA) may be employed as tentative diagnostic tools in order to identify anti-PaBV antibodies [37,38,39]. Unfortunately, there is no available commercial ELISA for PDD to date, and these tests are usually performed by few laboratories.

Although there seems to be a correlation between the level of antibody production and the development of clinical disease, it is important to note that some studies have demonstrated that many birds with positive fecal RT-PCR results were seronegative [38]. Additionally, birds that are clinically healthy may be seropositive. Therefore, serological assays must be interpreted carefully, and their utility, especially for decisions related to euthanasia, is limited [17].

## 3. Conclusions

The diagnosis of PDD must incorporate different diagnostic strategies for a successful outcome. For ante-mortem diagnosis, the combination of RT-PCR analysis of choanal and cloacal swabs from multiple timepoints with serological tests such as ELISA and diagnostic imaging techniques might yield reliable results. Post-mortem diagnosis should be guided by systematic necropsies followed by a thorough histopathological examination with confirmation by immunohistochemical and molecular analysis of selected tissues.

## Data Availability

The data that support the findings of this study are available from the corresponding author upon reasonable request.

## References

[1] Tizard I., Shivaprasad H.L., Guo J., Hameed S., Ball J., Payne S. (2016). The pathogenesis of proventricular dilatation disease. Anim. Health Res. Rev..

[2] Clark F.D. (1984). Proventricular Dilatation Syndrome in Large Psittacine Birds. Avian Dis..

[3] Honkavuori K.S., Shivaprasad H., Williams B.L., Quan P.-L., Hornig M., Street C., Palacios G., Hutchison S.K., Franca M., Egholm M. (2008). Novel Borna Virus in Psittacine Birds with Proventricular Dilatation Disease. Emerg. Infect. Dis..

[4] Kistler A.L., Gancz A., Clubb S., Skewes-Cox P., Fischer K., Sorber K., Chiu C.Y., Lublin A., Mechani S., Farnoushi Y. (2008). Recovery of divergent avian bornaviruses from cases of proventricular dilatation disease: Identification of a candidate etiologic agent. Virol. J..

[5] Piepenbring A.K., Enderlein D., Herzog S., Al-Ibadi B., Heffels-Redmann U., Heckmann J., Lange-Herbst H., Herden C., Lierz M. (2016). Parrot Bornavirus (PaBV)-2 isolate causes different disease patterns in cockatiels than PaBV-4. Avian Pathol..

[6] Heckmann J., Enderlein D., Piepenbring A.K., Herzog S., Heffels-Redmann U., Malberg S., Herden C., Lierz M. (2017). Investigation of Different Infection Routes of Parrot Bornavirus in Cockatiels. Avian Dis..

[7] Payne S., Shivaprasad H.L., Mirhosseini N., Gray P., Hoppes S., Weissenböck H., Tizard I. (2011). Unusual and severe lesions of proventricular dilatation disease in cockatiels (*Nymphicus hollandicus*) acting as healthy carriers of avian bornavirus (ABV) and subsequently infected with a virulent strain of ABV. Avian Pathol..

[8] Mirhosseini N., Gray P.L., Tizard I., Payne S. (2012). Complete Genome Sequence of Avian Bornavirus Genotype 1 from a Macaw with Proventricular Dilatation Disease. J. Virol..

[9] Gray P., Hoppes S., Suchodolski P., Mirhosseini N., Payne S., Villanueva I., Shivaprasad H., Honkavuori K.S., Briese T., Reddy S.M. (2010). Use of Avian Bornavirus Isolates to Induce Proventricular Dilatation Disease in Conures. Emerg. Infect. Dis..

[10] De Araújo J.L., Rech R.R., Heatley J.J., Guo J., Giaretta P.R., Tizard I., Rodrigues-Hoffmann A. (2017). From nerves to brain to gastrointestinal tract: A time-based study of parrot bornavirus 2 (PaBV-2) pathogenesis in cockatiels (*Nymphicus hollandicus*). PLoS ONE.

[11] Piepenbring A.K., Enderlein D., Herzog S., Kaleta E.F., Heffels-Redmann U., Ressmeyer S., Herden C., Lierz M. (2012). Pathogenesis of Avian Bornavirus in Experimentally Infected Cockatiels. Emerg. Infect. Dis..

[12] Kuhn J.H., Adkins S., Agwanda B.R., Al Kubrusli R., Alkhovsky S.V., Amarasinghe G.K., Avšič-Županc T., Ayllón M.A., Bahl J., Balkema-Buschmann A. (2021). 2021 Taxonomic update of phylum Negarnaviricota (Riboviria: Orthornavirae), including the large orders Bunyavirales and Mononegavirales. Arch. Virol..

[13] Amarasinghe G.K., Ayllón M.A., Bào Y., Basler C.F., Bavari S., Blasdell K.R., Briese T., Brown P.A., Bukreyev A., Balkema-Buschmann A. (2019). Taxonomy of the order Mononegavirales: Update 2019. Arch. Virol..

[14] Rossi G., Dahlhausen R.D., Galosi L., Orosz S.E. (2018). Avian Ganglioneuritis in Clinical Practice. Vet. Clin. N. Am. Exot. Anim. Pract..

[15] Boatright-Horowitz S.L. (2020). Avian Bornaviral Ganglioneuritis: Current Debates and Unanswered Questions. Vet. Med. Int..

[16] Deb A., Borjal R.J., Burkle M., Watson R., Hammer S. Evaluation of avian paramyxovirus.i serology and crop biopsy for the diagnosis of proventricular dilatation disease in captive Spix’s macaws (*Cyanopsitta spixii*). Proceedings of the European Association of Zoo- and Wildlite Velerinarians (EAZWV).

[17] Hoppes S., Gray P.L., Payne S., Shivaprasad H., Tizard I. (2010). The Isolation, Pathogenesis, Diagnosis, Transmission, and Control of Avian Bornavirus and Proventricular Dilatation Disease. Vet. Clin. N. Am. Exot. Anim. Pract..

[18] Philadelpho N.A., Rubbenstroth D., Guimarães M.B., Ferreira A.J.P. (2014). Survey of bornaviruses in pet psittacines in Brazil reveals a novel parrot bornavirus. Vet. Microbiol..

[19] De Araujo J.L., Hameed S., Tizard I., Escandon P., Giaretta P., Heatley J., Hoppes S., Rech R. (2019). Cardiac Lesions of Natural and Experimental Infection by Parrot Bornaviruses. J. Comp. Pathol..

[20] Gancz A.Y., Clubb S., Shivaprasad H. (2010). Advanced Diagnostic Approaches and Current Management of Proventricular Dilatation Disease. Vet. Clin. N. Am. Exot. Anim. Pract..

[21] Dennison S.E., Paul-Murphy J.R., Adams W.M. (2008). Radiographic determination of proventricular diameter in psittacine birds. J. Am. Vet. Med. Assoc..

[22] McMillan M.C., Ritchie B.W., Harrison G.J., Harrison L.R. (1999). Imaging techniques. Avian Medicine: Principles and Application.

[23] Hoppes S.M., Tizard I., Shivaprasad H. (2013). Avian Bornavirus and Proventricular Dilatation Disease. Vet. Clin. N. Am. Exot. Anim. Pract..

[24] Leal de Araujo J., Plumlee Q., Rech R.R., Winkel-Blair A., Hoppes S., Schneider S. (2016). Proventricular rupture associated with Psittaciforme 1 Bornavirus (PaBV) infection in a Major Mitchell Cockatoo (*Lophochroa leadbeateri*). Braz. J. Vet. Pathol..

[25] De Araujo J.L., Rodrigues-Hoffmann A., Giaretta P.R., Guo J., Heatley J., Tizard I., Rech R.R. (2018). Distribution of Viral Antigen and Inflammatory Lesions in the Central Nervous System of Cockatiels (*Nymphicus hollandicus*) Experimentally Infected with Parrot Bornavirus 2. Vet. Pathol..

[26] Smith D., Miller R.E., Lamberski N., Calle P.P. (2019). Bornaviruses in Birds. Fowler’s Zoo and Wild Animal Medicine Current Therapy.

[27] Gregory C.R., Latimer K.S., Campagnoli R., Ritchie B.W. (1996). Histologic Evaluation of the Crop for Diagnosis of Proventricular Dilatation Syndrome in Psittacine Birds. J. Vet. Diagn. Investig..

[28] Ouyang N., Storts R., Tian Y., Wigle W., Villanueva I., Mirhosseini N., Payne S., Gray P., Tizard I. (2009). Histopathology and the detection of avian bornavirus in the nervous system of birds diagnosed with proventricular dilatation disease. Avian Pathol..

[29] Rinder M., Ackermann A., Kempf H., Kaspers B., Korbel R., Staeheli P. (2009). Broad Tissue and Cell Tropism of Avian Bornavirus in Parrots with Proventricular Dilatation Disease. J. Virol..

[30] Weissenböck H., Bakonyi T., Sekulin K., Ehrensperger F., Doneley R.J., Dürrwald R., Hoop R., Erdélyi K., Gál J., Kolodziejek J. (2009). Avian Bornaviruses in Psittacine Birds from Europe and Australia with Proventricular Dilatation Disease. Emerg. Infect. Dis..

[31] Gancz A.Y., Kistler A.L., Greninger A.L., Farnoushi Y., Mechani S., Perl S., Berkowitz A., Perez N., Clubb S., DeRisi J.L. (2009). Experimental induction of proventricular dilatation disease in cockatiels (*Nymphicus hollandicus*) inoculated with brain homogenates containing avian bornavirus 4. Virol. J..

[32] Raghav R., Taylor M., DeLay J., Ojkic D., Pearl D.L., Kistler A.L., DeRisi J.L., Ganem D., Smith D.A. (2010). Avian Bornavirus is Present in Many Tissues of Psittacine Birds with Histopathologic Evidence of Proventricular Dilatation Disease. J. Vet. Diagn. Investig..

[33] Wünschmann A., Honkavuori K., Briese T., Lipkin W.I., Shivers J., Armien A.G. (2011). Antigen tissue distribution of Avian bornavirus (ABV) in psittacine birds with natural spontaneous proventricular dilatation disease and ABV genotype 1 infection. J. Vet. Diagn. Investig..

[34] Last R.D., Weissenböck H., Nedorost N., Shivaprasad H. (2012). Avian bornavirus genotype 4 recovered from naturally infected psittacine birds with proventricular dilatation disease in South Africa. J. S. Afr. Vet. Assoc..

[35] Hameed S.S., Guo J., Tizard I., Shivaprasad H., Payne S. (2018). Studies on immunity and immunopathogenesis of parrot bornaviral disease in cockatiels. Virology.

[36] Sigrist B., Geers J., Albini S., Rubbenstroth D., Wolfrum N. (2021). A New Multiplex Real-Time RT-PCR for Simultaneous Detection and Differentiation of Avian Bornaviruses. Viruses.

[37] Högemann C., Richter R., Korbel R., Rinder M. (2017). Plasma protein, haematologic and blood chemistry changes in African grey parrots (*Psittacus erithacus*) experimentally infected with bornavirus. Avian Pathol..

[38] Villanueva I., Gray P., Mirhosseini N., Payne S., Hoppes S., Honkavuori K.S., Briese T., Turner D., Tizard I. (2010). The diagnosis of proventricular dilatation disease: Use of a Western blot assay to detect antibodies against avian Borna virus. Vet. Microbiol..

[39] Sa-Ardta P., Rinder M., Sanyathitiseree P., Weerakhun S., Lertwatcharasarakul P., Lorsunyaluck B., Schmitz A., Korbel R. (2019). First detection and characterization of Psittaciform bornaviruses in naturally infected and diseased birds in Thailand. Vet. Microbiol..

